# PD-1 Blockage Reverses Immune Dysfunction and Hepatitis B Viral Persistence in a Mouse Animal Model

**DOI:** 10.1371/journal.pone.0039179

**Published:** 2012-06-22

**Authors:** Horng-Tay Tzeng, Hwei-Fang Tsai, Hsiu-Jung Liao, Yi-Jiun Lin, Lieping Chen, Pei-Jer Chen, Ping-Ning Hsu

**Affiliations:** 1 Graduate Institute of Immunology, College of Medicine, National Taiwan University, Taipei, Taiwan; 2 Department of Internal Medicine, Taipei Medical University, Shuang Ho Hospital, Taipei, Taiwan; 3 Institute of Clinical Medicine, College of Medicine, Taipei Medical University, Taipei, Taiwan; 4 Department of Microbiology, College of Medicine, National Taiwan University, Taipei, Taiwan; 5 Cancer Immunology Program at Yale Cancer Center, Yale University School of Medicine, New Haven, Connecticut, United States of America; 6 Graduate Institute of Clinical Medicine, College of Medicine, National Taiwan University, Taipei, Taiwan; 7 Department of Internal Medicine, National Taiwan University Hospital, Taipei, Taiwan; Singapore Institute for Clinical Sciences, Singapore

## Abstract

Persistent hepatitis B viral (HBV) infection results in chronic hepatitis, liver cirrhosis, and hepatocellular carcinoma (HCC). Recent studies in animal models of viral infection indicate that the interaction between the inhibitory receptor, programmed death (PD)-1, on lymphocytes and its ligand (PD-L1) play a critical role in T-cell exhaustion by inducing T-cell inactivation. High PD-1 expression levels by peripheral T-lymphocytes and the possibility of improving T-cell function by blocking PD-1-mediated signaling confirm the importance of this inhibitory pathway in inducing T-cell exhaustion. We studied T-cell exhaustion and the effects of PD-1 and PD-L1 blockade on intrahepatic infiltrating T-cells in our recently developed mouse model of HBV persistence. In this mouse animal model, we demonstrated that there were increased intrahepatic PD-1-expressing CD8+ and CD4+ T cells in mice with HBV persistence, but PD-1 upregulation was resolved in mice which had cleared HBV. The Intrahepatic CD8+ T-cells expressed higher levels of PD-1 and lower levels of CD127 in mice with HBV persistence. Blockade of PD-1/PD-L1 interactions increased HBcAg-specific interferon (IFN)-γ production in intrahepatic T lymphocytes. Furthermore, blocking the interaction of PD-1 with PD-L1 by an anti-PD-1 monoclonal antibody (mAb) reversed the exhausted phenotype in intrahepatic T lymphocytes and viral persistence to clearance of HBV *in vivo*. Our results indicated that PD-1 blockage reverses immune dysfunction and viral persistence of HBV infection in a mouse animal model, suggesting that the anti-PD-1 mAb might be a good therapeutic candidate for chronic HBV infection.

## Introduction

Hepatitis B virus (HBV) causes acute and chronic inflammatory liver diseases and subsequent hepatic cirrhosis and hepatocellular carcinoma (HCC). During chronic HBV infection, a dynamic balance between viral replication and the host immune response is pivotal to the pathogenesis of liver disease. It is widely accepted that adaptive immune responses, particularly cellular immune responses, mediate the clearance of HBV [1], [2] Unfortunately, HBV-specific T-cell function is impaired in patients with chronic HBV infection characterized by low levels of antiviral cytokines, impaired cytotoxic T lymphocyte activity, and persistent viremia [3]. However, the mechanism underlying this T-cell malfunction in chronic HBV infection is not completely understood.

The immunologic receptor, programmed death (PD)-1, a 55-kDa transmembrane protein containing an immunologic receptor tyrosine-based inhibitory motif, was originally isolated from a T-cell line exhibiting a high sensitivity to apoptosis [4]. The PD-1/PD-L1 pathway is well documented to play a negative role in regulating activation and proliferation of T-cells and production of cytokines [5], [6]. There is evidence that the PD-1 pathway plays an important role in inhibiting the function of virus-specific CD8+ T-cells in chronic viral infection involving human immunodeficiency virus (HIV) [7], hepatitis C virus (HCV) [8], and HBV [9]. Although reports are available on changes in expression levels of PD-1 and T-cell responses in patients with HBV infection, the pattern of change of PD-1 expression in the natural course of chronic HBV infection has not yet been presented.

**Figure 1:**
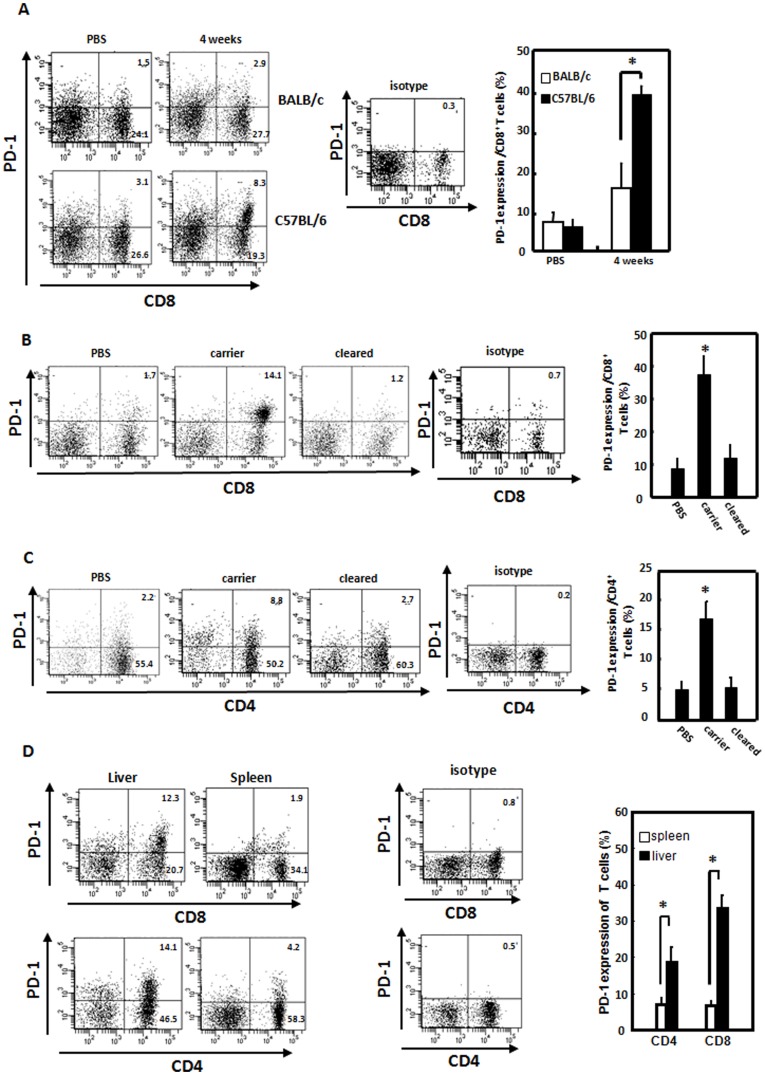
Increased programmed death (PD)-1-expressing CD8+ and CD4+ T-cells in liver-infiltrating lymphocytes from mice with hepatitis B viral (HBV) persistence. A. C57BL/6 or BALB/c mice were hydrodynamically injected with the pAAV/HBV1.2 plasmid. Four weeks post-injection, intrahepatic lymphocytes were isolated and PD-1 expression was analyzed by flow cytometry. B and C. C57BL/6 mice were hydrodynamically injected with pAAV/HBV1.2. Four weeks after hydrodynamic injection, intrahepatic lymphocytes from mice which were HBsAg-positive (carrier) or HBsAg-negative (cleared) were isolated, and the PD-1 expressions by CD8^+^ (B) and CD4^+^ (C) T-ells were analyzed by flow cytometry. The data were representative of at least 12 independent experiments. D. C57BL/6 mice were injected with pAAV/HBV1.2 plasmid hydrodynamically. HBsAg-positive carrier were sacrificed at 4 weeks post-injection. Intrahepatic lymphocytes and splenocytes were isolated and the populations of CD4^+^PD-1^+^ or CD8^+^PD-1^+^ were determined by flow cytometry analysis. *p<0.05. The data were representative of at least 6 independent experiments.

Recent studies using animal models of viral infection in both mice and macaques indicated that the interaction between the inhibitory PD-1 on lymphocytes and its ligand (PD-L1) plays a critical role in T-cell exhaustion by inducing T-cell inactivation, and high PD-1 expression levels by peripheral T-lymphocytes and the possibility of improving T-cell function by blocking PD-1-mediated signaling confirm the importance of this inhibitory pathway in inducing T-cell exhaustion [10], [11], [12]. Moreover, *in vivo* manipulation of costimulatory pathways to restore the antiviral function of exhausted T-cells was successfully applied in mice persistently infected with a lymphocytic choriomeningitis virus to improve therapeutic vaccinations [13]. Also, in various human chronic infections, including hepatitis B, high PD-1 levels are expressed by virus-specific T-cells, and improved T-cell function was obtained *in vitro* by inhibiting the PD-1/PD-L1 interaction. We recently developed a mouse model of HBV persistence, in which a single intravenous (i.v.) hydrodynamic injection of HBV DNA to C57BL/6 mice allows HBV replication and induces a partial immune response, so that about 40% of the mice carry HBV for more than 6 months [14]. The model was used to identify the viral antigen crucial for HBV persistence. Our study indicated that knocking out HBcAg, but not HBeAg or pol, led to HBV persistence in mice, and the essential region of HBcAg was the carboxyl terminus, specifically the 10 terminal amino acids (HBcAg176∼185) [15]. These results indicate that the immune response triggered in mice by HBcAg during exposure to HBV is important in determining HBV persistence. Tolerance toward the HBV surface antigen in this model was shown to be due to insufficient cellular immunity against the hepatitis B core antigen, as was documented in humans.

This study was thus undertaken to further define the role of T-cell exhaustion in chronic HBV infection in a mice animal model, by comparing the phenotype and function of intrahepatic infiltrating T lymphocytes in mice with HBV persistence or HBV clearance, and the effect of PD-1/PD-L1 blockade in restoring immune dysfunction and clearance of HBV. It is the first report to demonstrate PD-1/PD-L1 blockade could reverses immune dysfunction and HBV viral persistence *in vivo*. This observation opens new potential perspectives for the development of novel immunotherapies for chronic hepatitis B.

**Figure 2:**
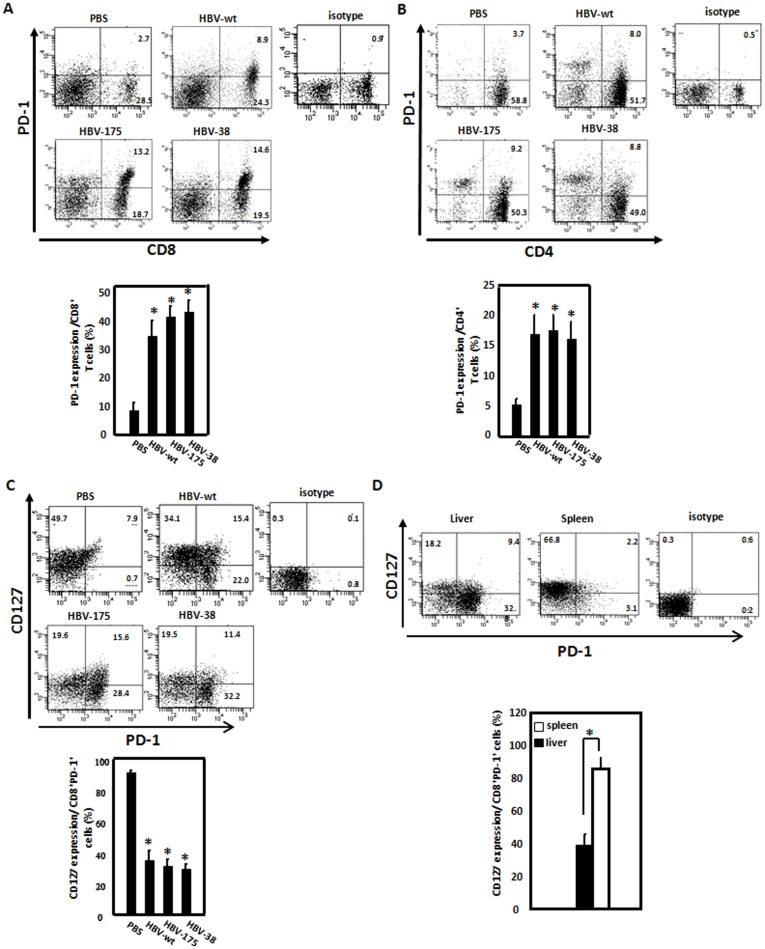
Liver-infiltrating CD8+ lymphocytes in carrier mice displayed the PD-1^hi^CD127^low^-exhausted phenotype. A and B. HBV core mutants induced upregulation of PD-1 expression in intrahepatic T lymphocytes. C57BL/6 mice were hydrodynamically injected with WT pAAV/HBV1.2 or HBV core mutant DNA, including HBV-175 or HBV-38 constructs. Four weeks after the injection, intrahepatic lymphocytes were isolated, and the PD-1 expressions by CD8^+^ (A) and CD4^+^ (B) T-cells were analyzed by flow cytometry. C. C57BL/6 mice were hydrodynamically injected with wild type hepatitis B virus (HBV-wt) and HBV core mutants. Intrahepatic lymphocytes were isolated at 4 weeks, and PD-1 and CD127 expressions by CD8^+^ T cells were analyzed by flow cytometry. D. C57BL/6 mice were hydrodynamically injected with HBV core mutant DNA, HBV-175. Intrahepatic infiltrating lymphocytes and splenic lymphocytes were isolated at 4 weeks, and PD-1 and CD127 expressions by CD8^+^ T cells were analyzed by flow cytometry. *p<0.05. The data were representative of at least 6 independent experiments.

**Figure 3:**
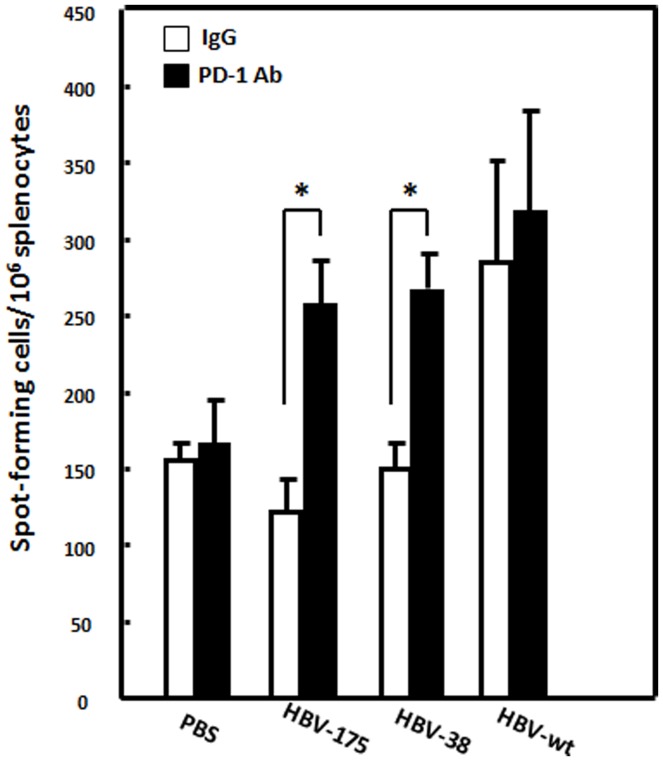
Impaired HBcAg-specific interferon (IFN)-γ T-cell response in C57BL/6 mice with hepatitis B viral (HBV) persistence was reversed by treatment with anti-PD-1 mAb. C57BL/6 mice were hydrodynamically injected with WT pAAV/HBV1.2 or HBV core mutant DNA, including HBV-175 or HBV-38 constructs. Ten days after the injection, splenocytes were isolated and HBcAg-specific IFN-γ responses were analyzed by an ELISpot assay. The frequency of HBcAg-specific IFN-γ-secreting cells in the presence of an anti-PD-1 or control antibody were measured. Spot-forming cells per million splenocytes are shown. *p<0.05.

**Figure 4:**
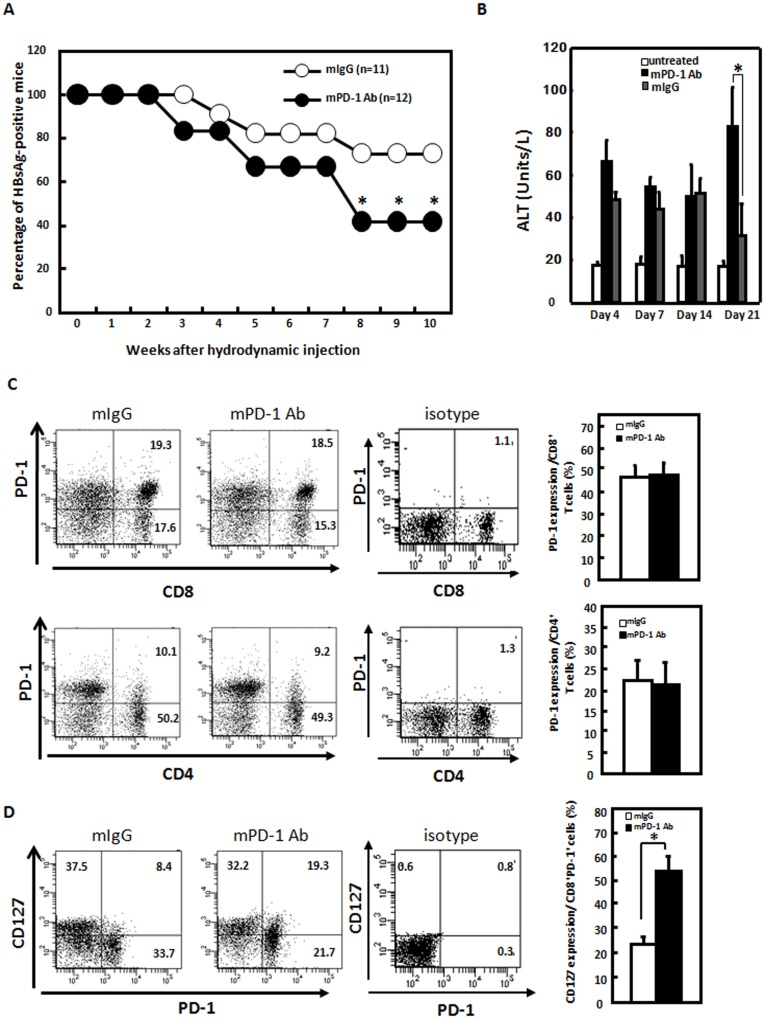
Blockade of the programmed death (PD)-1 pathway by an anti-PD-1 monoclonal antibody (mAb) reduced the hepatitis B viral (HBV) persistence rate and reversed PD-1^hi^CD127^low^-exhausted CD8+ T cells phenotype in a mouse animal model. A. C57BL/6 mice were intraperitoneally treated with an anti-PD-1 blocking mAb or isotype control Ab. Antibody administration was initiated every 2 days on day 6 before being injected with an HBV core-truncated (HBV-175) mutant construct. Thereafter, Abs (200 µg) were repeatedly administered every 3∼4 days for a period of 8 weeks. The rates of positive serum HBsAg in the mice receiving anti-PD-1 blocking mAb (•, n = 12) were compared with those in mice receiving isotype control Ab (○, n = 11), The HBsAg level in mice serum was determined by an ELISA. HBsAg-positive mice were defined as having a signal-to-noise (S/N) ratio of ≥2. B. The ALT levels in the mice receiving anti-PD-1 blocking mAb, isotype control Ab, and untreated. The ALT level in mice serum was determined by an ELISA. *p<0.05. C and D. Increased CD127 expression and reversed PD-1^hi^CD127^low^-exhausted CD8+ T cells phenotype in mice treated with an anti-PD-1 mAb. Intrahepatic lymphocytes from C57BL/6 mice hydrodynamically injected with wild-type (WT) pAAV/HBV1.2 and HBsAg-positive 4 weeks after the injection were isolated, and the expressions of PD-1 (C) as well as coexpression of PD-1 and CD127 (D) on CD4+ and CD8+ T cells in the presence of an anti-PD-1 or control antibody were measured by flow cytometry. Serum HBsAg titers were determined by an ELISA. HBsAg-positive mice were defined as having a signal-to-noise (S/N) ratio of ≥2. *p<0.05.

## Materials and Methods

### Animals and HBV Constructs

Male C57BL/6 and BALB/c mice were obtained from the Animal Center of National Taiwan University and maintained under specific pathogen-free (SPF) conditions. All animal experiments were performed according to regulations approved by the Animal Ethical Committee of National Taiwan University. The HBV constructs and pAAV/HBV1.2 mutants were generated by site-directed mutagenesis as previously described [14], [15]. Briefly, site-directed mutagenesis was used to generate pAAV/HBV1.2 mutants with a QuickChang II site-directed mutagenesis kit (Stratagene, La Jolla, CA) following the manufacturer’s instructions. Then, the mutants were amplified by a polymerase chain reaction (PCR) using pAAV/HBV1.2 as a template.

### Hydrodynamic Injection

C57BL/6 mice and BALB/c (male, 6∼7 weeks old) were anesthetized with ketamine and xylazine. Ten micrograms of HBV plasmid DNA in a volume equivalent to 8% of the mouse body weight was injected via a tail vein in 5 s. Serum HBsAg, HBeAg, anti-HBs, and anti-HBc levels were assayed as indicated to monitor the state of HBV persistence.

### Isolation of Intrahepatic Leukocytes

Livers were perfused with 0.2% bovine serum albumin (BSA)/phosphate-buffered saline (PBS), passed through a nylon mesh, and digested in collagense-IV and DNase-I (Sigma-Aldrich, St. Louis, MO) for 30 min. Hepatocytes were removed by centrifugation for 5 min at 100 *g* and washed with 0.2% BSA/PBS twice at 500 *g* for 5 min. Leukocyte subsets were isolated by using OptiPrep® Density Gradient Medium (Sigma-Aldrich, St. Louis, MO).

### Detection of the HBV Antigen, Antibody (Ab), and DNA

Serum levels of HBsAg, HBeAg, anti-HBc, and anti-HBs Abs were determined using the AXSYM system kit (Abbott Diagnostika, Wiesbaden, Germany). The cutoff value for determining HBsAg positivity was a signal-to-noise (S/N) ratio of ≥2 and a signal-to-cutoff (S/CO) ratio of ≥1. To detect serum HBV DNA, each serum sample was pretreated with 25 units of DNase I (Roche Diagnostics, Mannheim, Germany) at 37°C overnight, and total DNA was extracted and HBV DNA was detected by a real-time PCR as previously described [14], [15]. Serum alanine transferase (ALT) was measured on a TBA-200FR automated clinical chemistry analyzer (Toshiba, Tokyo, Japan) as previously described [14].

### Interferon (IFN)-γ Enzyme-linked Immunospot (ELISpot) Assay

At indicated time points after the hydrodynamic injection, mice were killed, and splenocytes were cultured and assayed for the frequencies of antigen-specific IFN-γ- secreting cells using an ELISPOT kit (BD Biosciences, San Jose, CA). Briefly, 10^6^ splenocytes were co-cultured with 1 µg/ml of rHBcAg (ID Labs, London, Canada) in 200 µl RPMI 1640 supplemented with 10% fetal calf serum (FCS). Cell suspensions were incubated for 20 h. Spot-forming cells were revealed with a biotin-conjugated antibody, streptavidin-horseradish peroxidase (HRP) and AEC substrates (Sigma-Aldrich) and were analyzed using the ImmunoSpot series 5 analyzer (Cellular Technology Limited, Cleveland, OH).

### Flow Cytometry

For flow cytometry, allophycocyanin (APC)-conjugated anti-mouse CD3 (BD Biosciences, Palo Alto, CA), phycoerythrin (PE)-conjugated anti-mouse PD-1, fluorescein isothiocyanate (FITC)-conjugated CD127, and PE-Cy5.5-conjugated anti-mouse CD4 or CD8 (BD Biosciences Pharmingen) monoclonal (m)Abs were used for flow cytometry. For the flow cytometric analysis, 10^5^ cells were labeled in a fluorescence-activated cell sorter (FACS) buffer (PBS/2% FCS/0.1% sodium azide), fixed in 1% paraformaldehyde (Sigma-Aldrich, St. Louis, MO) and analyzed on a FACSCalibur using the CellQuest software (Becton Dickinson, Mountain View, CA).

### In vivo Treatment of Mice with the Anti-PD-1 mAb

An anti mouse PD-1 Ab was purified from hybridoma (clone G4) culture supernatant (kindly provided by Dr. Lieping Chen, Yale University). C57BL/6 mice were intraperitoneally (i.p.) treated with an anti-PD-1 blocking Ab or isotype control Ab. Ab administration was initiated every 2 days on day 6 before injection with the HBV core-truncated (HBV-175) mutant construct. Thereafter, Abs (200 µg) were repeatedly administered every 3∼4 days for a period of 8 weeks.

## Results

### Increased PD-1-expressing CD8+ and CD4+ T-cells in Liver-infiltrating Lymphocytes from Mice with HBV Persistence

Although the chronicity of HBV infection is the result of impaired HBV-specific immune responses that cannot efficiently eliminate or cure infected hepatocytes, many issues remain unsettled. We established a mouse model of HBV persistence in immunocompetent mice by a hydrodynamic injection of replication-competent HBV DNA. This approach generated HBV persistence in C57BL/6 mice, but not in BALB/c mice [14], [15]. C57BL/6 and BALB/c mice were hydrodynamically injected with the pAAV/HBV1.2 plasmid. At the indicated time points, intrahepatic lymphocytes from mice that were HBsAg-positive (carrier) or HBsAg-negative (cleared) were isolated, and PD-1 expressions by CD8+ and CD4+ T-cells were analyzed by flow cytometry. The results in Figure 1 demonstrate that there were increased proportions of PD1-expressing CD8+ and CD4+ T-cells in livers of C57BL/6 mice, but not in BALB/c mice. The PD-1 upregulation was not observed in splenic lymphocytes in C57BL/6 mice. Moreover, in C57BL/6 mice, both PD-1-expressing CD8+ and CD4+ intrahepatic T-cells significantly decreased in mice which had cleared the injected pAAV/HBV1.2 plasmid (cleared mice), compared to mice with HBV persistence (carrier mice) (Figure 1). These results indicate a significant increase in PD-1 expression in both intrahepatic CD8+ and CD4+ T-cells of mice with HBV persistence. This is consistent with the results of recent studies of HBV-infected patients, which demonstrated that PD-1 expression can impair virus-specific CD8 T-cell responses during chronic HBV infection [16], [17], [18], [19], [20].

### Liver-infiltrating CD8+ Lymphocytes in Carrier Mice Displayed the PD-1^hi^CD127^low^-exhausted Phenotype

Furthermore, to further define the role of the HBV core in HBV persistence in this mice animal model, C57BL/6 mice were injected with wild-type (WT) pAAV/HBV1.2 or HBV core mutant DNA, including HBV-175 or HBV-38 [15], and HBV persistence and PD-1 expression in liver-infiltrating lymphocytes were analyzed by flow cytometry. Both the HBV-175 and HBV-38 core-mutant viral constructs caused significant loss of the ability to raise the host immunity to clear the virus, with increased PD-1-expressing T-cell infiltration, leading to HBV persistence in mice (Figure 2), consistent with the observation that the HBV core protein plays an important role in induction of a host immune response to HBV [15].

Recent studies in animal models of viral infection indicated that the interaction between PD-1 on lymphocytes and its ligands plays a critical role in T-cell exhaustion by inducing T-cell inactivation. HBV-specific PD-1-positive CD8 cells of patients with chronic HBV infection also displayed lower levels of the interleukin (IL)-7 receptor, CD127, which was previously described in association with the exhausted phenotype [9], [21]. To define the exhausted phenotype in the PD-1-expressing intrahepatic T-cells in mice with HBV persistence, liver-infiltrating CD8+ lymphocytes from mice hydrodynamically injected with the HBV construct were evaluated for the expressions of both PD-1 and CD127 by CD8^+^ T-cells by flow cytometry (Figure 2). The results demonstrate that PD-1 was more highly expressed by intrahepatic CD8 cells in mice with HBsAg persistence. Also, among intrahepatic CD8 populations, CD127 expression was significantly lower in carrier mice compared to mice which had cleared HBV. The PD-1^hi^CD127^low^-exhausted phenotype was not observed in splenic lymphocytes in mice with HBsAg persistence. Our results indicate that liver-infiltrating CD8+ lymphocytes in carrier mice displayed the PD-1^hi^CD127^low^-exhausted phenotype.

We then asked whether the PD-1/PD-L1 interaction of intrahepatic T-cells was associated with the impaired immune response, resulting in a defective T-cell response to HBV in mice with HBV persistence after an infection. The HBV core-specific IFN-γ T-cell response in mice hydrodynamically injected with WT pAAV/HBV1.2 (HBV-wt), HBV-175, or HBV-38 in the presence and absence of anti-PD-1 mAb treatment were analyzed by an ELISpot assay. Results in Figure 3 demonstrate that the frequency of HBcAg-specific IFN-γ-secreting cells was significantly reduced in mice injected with HBV core mutant constructs; however, impairment of the HBcAg-specific IFN-γ response was restored when treated with the anti-PD-1 mAb. Taken together, our results indicate that the impaired T-cell response to the HBV core in the IFN-γ ELISpot assay could be restored by treatment with an anti-PD-1 mAb, indicating that the level of PD-1 expression on intrahepatic T cells is correlated with the anti-viral T-cell response *in vivo*.

### Blockade of the PD-1 Pathway by an Anti-PD-1 mAb Reduced the HBV Persistence Rate in this Mouse Animal Model

To further define the role of T-cell exhaustion in the pathogenesis of persistent HBV infection in this mice animal model, and determine the effect of PD-1/PD-L1 blockade on restoring immune dysfunction and clearance of HBV, C57BL/6 mice were intraperitoneally treated with an anti-PD-1 blocking mAb or a control isotype Ab. Ab administration was initiated every 2 days on day 6 before an injection with the HBV core-truncated (HBV-175) mutant construct. Thereafter, Abs (200 µg) were repeatedly administered every 3∼4 days for a period of 8 weeks. The HBsAg level in mice serum was determined by an ELISA. The results in Figure 4 demonstrated that blocking the interaction of PD-1/PD-L1 by the anti-PD-1 mAb significantly reduced the frequency of HBV persistence in mice injected with the core-null HBV viral construct, resulting in clearance of HBV *in vivo*. The mice treated with anti-PD-1 mAb with higher viral clearance rate and also showed higher ALT level compared to control mice. In mice treated with anti-PD-1 mAb, the PD-1 expression level in liver infiltrating lymphocytes after hydrodynamic infection of HBV was similar to mice treated with control Ig. In contrast, among intrahepatic CD8 populations, CD127 expression was significantly higher in mice treated anti-PD-1 mAb compared to mice treated with control Ig (Figure 4), indicating PD-1 blockage reversed PD-1^hi^CD127^low^-exhausted phenotype in intrahepatic CD8+ T cells in mice treated with anti-PD-1 mAb. Taken together, these results indicate that PD-1 blockage reverses immune dysfunction as well as the PD-1^hi^CD127^low^-exhausted phenotype and viral persistence to HBV infection in this mouse animal model, suggesting that the anti-PD-1 mAb might be a good therapeutic candidate for chronic HBV infection.

## Discussion

In this study, we demonstrated that there were increased intrahepatic PD-1-expressing CD8+ and CD4+ T-cells in mice with HBV persistence. Intrahepatic-infiltrating CD8+ T-cells expressed higher levels of PD-1 and lower levels of CD127 in mice with HBV persistence. Furthermore, PD-1/PD-L1 blockade partially restored the function of intrahepatic T-cells, leading to viral clearance. Although there were studies with similar observations from HBV transgenic mice [11] and clinical subjects [16], [19], [22], it is the first report to demonstrate PD-1/PD-L1 blockade could reverses immune dysfunction and HBV viral persistence *in vivo*. Moreover, even though reports are available on changes in expression levels of PD-1 and T-cell responses in patients with HBV infection, the pattern of change of PD-1 expression in the course of HBV infection *in vivo* has not yet been presented. The results from our study open new potential perspectives for the development of novel immunotherapies for chronic hepatitis B.

Our results also demonstrated there were increased PD1-expressing CD8+ and CD4+ T-cells in mice infected with core mutants with viral persistence, indicating the HBV core protein, in particular the terminal 10 amino acids, is critical for inducing immunity to HBV. In a previous study, we isolated liver-infiltrating lymphocytes (IHL) from liver tissues of chronic hepatitis B patients and analyzed their immune phenotype. We found a significantly increased CD8^+^ T-cell population with increased PD-1 and decreased CD28 [20]. These data are consistent with the findings in this mouse model and suggest a tolerant immune response underlying chronic HBV infection in both humans and mice. Furthermore, the impaired T-cell response to the HBV core in the IFN-γ ELISpot assay was restored by treatment with the anti-PD-1 mAb (Figure 3), indicating that PD-1 expression is correlated with the *in vivo* antiviral T-cell response. This is consistent with a recent report that antiviral intrahepatic T-cell responses could be restored by blocking the PD-1 pathway *ex vivo* in patients with chronic hepatitis B infection [22].

Studies in animal models of viral infections show that persistent exposure to high antigen concentrations can affect the antiviral T-cell function by causing different degrees of functional impairment, up to physical T-cell deletion, as a function of the quantity of antigens to which T-cells are exposed [23]. T-cell exhaustion by this mechanism may play an important role in the pathogenesis of HBV-specific T-cell hyporesponsiveness with chronic HBV infection, because high antigen concentrations are consistently present at all stages of the infection. The importance of T-cell exhaustion in chronic HBV infection was confirmed by the high expression of PD-1 by circulating HBV-specific CD8 cells and by the possibility of partially improving the peripheral blood T-cell function by blocking the interaction of PD-1/PD-L1 with anti-PD-L1 Abs [9]. Moreover, additional support for exhaustion is provided by the inverse correlation between the function of HBV-specific T-cells and viremia levels, since T-cells are more profoundly inhibited in the presence of high viremia [9], [24]. Because T-cells are an essential component of antiviral protection, restoration of efficient T-cell function may represent a strategy to cure infections, and manipulation of costimulatory pathways involved in T-cell activation may be a rational approach to achieve this goal. The results obtained in this study that PD-1 blockage reverses immune dysfunction as well as the PD-1^hi^CD127^low^-exhausted phenotype, and viral persistence to HBV infection in this mouse animal model further support this notion.

The outcome of HBV infection, recovery or persistence, is controlled by the interplay between viral proteins and host factors. The genetic background of recipients, which correlates with the strength of immune responses against HBV antigens during primary activation, also determines the outcome after hydrodynamic injection. In our results, the CD8+ T cells from BALB/c mice had significant lower PD-1 expression compared to C57BL/6 mice. Moreover, in C57BL/6 mice, the PD-1-expressing CD8+ intrahepatic T-cells significantly decreased in mice which had cleared the injected HBV DNA (cleared mice), compared to mice with HBV persistence (carrier mice). These results indicate a significant increase in PD-1 expression in intrahepatic T cells of mice with HBV persistence. In HBV carrier C57BL/6 mice, impaired HBcAg-specific immune responses failed to clear HBV DNA [14]. Yang et al. [25] have observed persistent expression of HBV antigens in the hepatocytes of immunocompromised mice after hydrodynamic injection of HBV plasmid DNA. These data also suggested that impaired immune responses in injected mice would allow input HBV DNA persistence and increased PD-1 expression in intrahepatic T cells. Our nontransgenic mouse model for HBV persistence provides opportunities to investigate the mechanisms and host factors determining HBV persistence.

In conclusion, in this study, we demonstrated that blockade of the PD-1/PD-L1 interaction increased IFN-γ production in response to the HBV core by intrahepatic lymphocytes. Furthermore, blocking the interaction of PD-1 with its ligand, PD-L1, by an anti-PD-1 mAb reversed the viral persistence to clearance of the core-null HBV viral construct in this mouse animal model. Our results indicate that PD-1 blockage reverses immune dysfunction and viral persistence to HBV infection in this mouse animal model, suggesting that anti-PD-1 might be a good therapeutic candidate for chronic HBV infection.

## References

[1] Chisari FV, Ferrari C (1995). Hepatitis B virus immunopathogenesis.. Annu Rev Immunol.

[2] Jung MC, Pape GR (2002). Immunology of hepatitis B infection.. Lancet Infect Dis.

[3] Iwai Y, Terawaki S, Ikegawa M, Okazaki T, Honjo T (2003). PD-1 inhibits antiviral immunity at the effector phase in the liver.. J Exp Med.

[4] Ishida Y, Agata Y, Shibahara K, Honjo T (1992). Induced expression of PD-1, a novel member of the immunoglobulin gene superfamily, upon programmed cell death.. Embo J.

[5] Chen L (2004). Co-inhibitory molecules of the B7-CD28 family in the control of T-cell immunity.. Nat Rev Immunol.

[6] Nurieva R, Thomas S, Nguyen T, Martin-Orozco N, Wang Y (2006). T-cell tolerance or function is determined by combinatorial costimulatory signals.. Embo J.

[7] Day CL, Kaufmann DE, Kiepiela P, Brown JA, Moodley ES (2006). PD-1 expression on HIV-specific T cells is associated with T-cell exhaustion and disease progression.. Nature.

[8] Golden-Mason L, Palmer B, Klarquist J, Mengshol JA, Castelblanco N (2007). Upregulation of PD-1 expression on circulating and intrahepatic hepatitis C virus-specific CD8+ T cells associated with reversible immune dysfunction.. J Virol.

[9] Boni C, Fisicaro P, Valdatta C, Amadei B, Di Vincenzo P (2007). Characterization of hepatitis B virus (HBV)-specific T-cell dysfunction in chronic HBV infection.. J Virol.

[10] Barber DL, Wherry EJ, Masopust D, Zhu B, Allison JP (2006). Restoring function in exhausted CD8 T cells during chronic viral infection.. Nature.

[11] Maier H, Isogawa M, Freeman GJ, Chisari FV (2007). PD-1:PD-L1 interactions contribute to the functional suppression of virus-specific CD8+ T lymphocytes in the liver.. J Immunol.

[12] Velu V, Titanji K, Zhu B, Husain S, Pladevega A (2009). Enhancing SIV-specific immunity in vivo by PD-1 blockade.. Nature.

[13] Ha SJ, Mueller SN, Wherry EJ, Barber DL, Aubert RD (2008). Enhancing therapeutic vaccination by blocking PD-1-mediated inhibitory signals during chronic infection.. J Exp Med.

[14] Huang LR, Wu HL, Chen PJ, Chen DS (2006). An immunocompetent mouse model for the tolerance of human chronic hepatitis B virus infection.. Proc Natl Acad Sci U S A.

[15] Lin YJ, Huang LR, Yang HC, Tzeng HT, Hsu PN (2010). Hepatitis B virus core antigen determines viral persistence in a C57BL/6 mouse model.. Proc Natl Acad Sci U S A.

[16] Zhang Z, Zhang JY, Wherry EJ, Jin B, Xu B (2008). Dynamic programmed death 1 expression by virus-specific CD8 T cells correlates with the outcome of acute hepatitis B. Gastroenterology 134: 1938–1949, 1949 e1931–1933..

[17] Zhang Z, Jin B, Zhang JY, Xu B, Wang H (2009). Dynamic decrease in PD-1 expression correlates with HBV-specific memory CD8 T-cell development in acute self-limited hepatitis B patients.. J Hepatol.

[18] Yang PL, Althage A, Chung J, Maier H, Wieland S (2010). Immune effectors required for hepatitis B virus clearance.. Proc Natl Acad Sci U S A.

[19] Xie Z, Chen Y, Zhao S, Yang Z, Yao X (2009). Intrahepatic PD-1/PD-L1 up-regulation closely correlates with inflammation and virus replication in patients with chronic HBV infection.. Immunol Invest.

[20] Hsu PN, Yang TC, Kao JT, Cheng KS, Lee YJ (2010). Increased PD-1 and decreased CD28 expression in chronic hepatitis B patients with advanced hepatocellular carcinoma.. Liver Int.

[21] Boettler T, Panther E, Bengsch B, Nazarova N, Spangenberg HC (2006). Expression of the interleukin-7 receptor alpha chain (CD127) on virus-specific CD8+ T cells identifies functionally and phenotypically defined memory T cells during acute resolving hepatitis B virus infection.. J Virol.

[22] Fisicaro P, Valdatta C, Massari M, Loggi E, Biasini E (2010). Antiviral intrahepatic T-cell responses can be restored by blocking programmed death-1 pathway in chronic hepatitis B. Gastroenterology 138: 682–693, 693 e681–684..

[23] Wherry EJ, Ha SJ, Kaech SM, Haining WN, Sarkar S (2007). Molecular signature of CD8+ T cell exhaustion during chronic viral infection.. Immunity.

[24] Maini MK, Boni C, Lee CK, Larrubia JR, Reignat S (2000). The role of virus-specific CD8(+) cells in liver damage and viral control during persistent hepatitis B virus infection.. J Exp Med.

[25] Yang PL, Althage A, Chung J, Chisari FV (2002). Hydrodynamic injection of viral DNA: a mouse model of acute hepatitis B virus infection.. Proc Natl Acad Sci U S A.

